# Exploring cultural determinants to be integrated into preterm infant care in the neonatal intensive care unit: an integrative literature review

**DOI:** 10.1186/s12884-022-05321-7

**Published:** 2023-01-09

**Authors:** Madimetja Nyaloko, Welma Lubbe, Salaminah S. Moloko-Phiri, Khumoetsile D. Shopo

**Affiliations:** NuMIQ Research Focus Area, North-West University, Potchefstroom, South Africa

**Keywords:** Cultural determinants, Culturally sensitive care, Neonatal intensive care unit, Parents; preterm infants

## Abstract

**Background:**

Cultural practices are an integral part of childrearing and remain a significant aspect for healthcare professionals to ensure culturally sensitive care, particularly in the neonatal intensive care unit.

**Objective:**

To synthesise literature on the cultural determinants that can be integrated into care of preterm infants admitted into the neonatal intensive care unit.

**Methods:**

The current review followed the integrative literature review steps proposed by Lubbe and colleagues. The registration of the review protocol was in PROSPERO**.** There was a literature search conducted in the EBSCOhost, PubMed, ScienceDirect and Scopus databases using the search string developed in collaboration with the librarian. Three reviewers employed a three-step screening strategy to screen the articles published in English between 2011 and 2021 that focused on culturally sensitive care. The Johns Hopkins Nursing Evidence-Based Practice Evidence critical appraisal toolkit assessed the methodological quality of the articles included at the full-text screening level.

**Results:**

There were 141 articles retrieved, and 20 included on the full-text screening level; the exclusion of one article was due to a low critical appraisal grade. Four topical themes emerged from 19 articles: spiritual care practices, intragenerational infant-rearing practices, infant physical care practices, and combining treatment practices.

**Conclusion:**

Overall, the findings indicated that parental cultural beliefs and practices mostly influenced infant-rearing practices, emphasising the significance of integrating cultural practices when rendering healthcare services. The recommendation is that healthcare professionals understand various cultural determinants, mainly those specific to the community they serve, to provide culturally sensitive care.

**Supplementary Information:**

The online version contains supplementary material available at 10.1186/s12884-022-05321-7.

## Introduction and background

Globally, people have specific cultural practices related to nutrition, education, and health, including childrearing. Culture plays a significant role in all spheres of healthcare. Cultural practices, as an integral part of childrearing, remain a significant aspect that healthcare professionals should consider to ensure culturally sensitive care to infants and parents, particularly in the neonatal intensive care unit (NICU) [1]. The search for a universally accepted definition of culture has lasted for decades [2]. Culture is a multifaceted concept that encompasses more than just people’s daily routines and refers to the country’s associated or defined collection of commonly held features, such as traditional beliefs, values, and social practices, which influence the lives of the community [3–5]. Furthermore, culture is a complex social matrix characterised by “cultural determinants,” which refer to the features or factors that primarily distinguish one culture from another and shape ways of perceiving and accessing the world [3].

Cultural beliefs and practices influence how parents use healthcare services and interact with healthcare professionals. A study conducted in Ghana has shown the significant relationship between the cultural practices, norms, beliefs, and behaviours that influence maternal and child healthcare [6]. Furthermore, covert cultural practices are more likely to have detrimental health consequences when taken for granted [7]. Consequently, there is a need to understand the cultural practices, and beliefs, influencing maternal and child healthcare in hospital settings to enhance culturally sensitive healthcare services.

Childrearing in a cultural context is a unique practice, according to specific cultural customs and norms guiding parents and families’ behaviours [8]. Parental transformation and parent-infant interactions are dynamic, and the processes become complicated when there are preterm infants involved. Furthermore, parent-infant interaction is primarily shaped by specific cultural practices, beliefs, and caregiving contexts [8].

Preterm infants are babies born alive before the completion of the 37 weeks of pregnancy [9]. Health conditions of preterm infants often require hospital admission in neonatal intensive care units and later neonatal wards [10]. Furthermore, care of preterm infants during hospitalisation requires parental involvement [11].

Although studies have demonstrated the importance of integrating cultural practices into healthcare services, there has been no formal process of integrating cultural practices or beliefs into neonatal care. Furthermore, no study has explored and comprehensively synthesised the literature on the possible integration of cultural determinants into preterm infant care in the NICU. Thus, this paper aims to conduct an integrative literature review to synthesise whether there could be cultural determinants to be integrated into the care of preterm infants in the NICU as an intervention to ensure and promote culturally sensitive care.

## Materials and methods

The conducting of the current integrative literature review was in accordance with a registered and published protocol on PROSPERO, Reg. No: CRD42021283895 [12]. In addition, the review adhered to a rigorous set of five steps for integrative review by Lubbe et al. [13], as deduced from authors such as Whittemore and Knafl [14], Toracco [15], Rusells [16], de Souza et al. [17]

### Composition of review question

The PIOS model composed the review question [18]. PIOS is the mnemonic for the population (P), phenomena of interest (I), outcome (O) and setting (S) [19, 20]. In the context of the current review, P stands for the parent of preterm infants, I-cultural determinants, O-culturally sensitive care, and S-neonatal intensive care unit. The review question was, therefore, what is the best available published evidence on cultural determinants that could be integrated into preterm infant care in the NICU to ensure culturally sensitive care?

### Sampling literature

Sampling of the literature was conducted in two steps, searching and screening, as described below.

#### Searching (scoping search and search strategy development)

The preliminary databases and PROSPERO searches revealed no previous or current reviews on the same topic. The search in EBSCOhost, PubMed, ScienceDirect, and Scopus databases was for published articles in September 2021 and again in October 2021 for updates. A comprehensive search was conducted in conjunction with the North-West University (NWU) librarian [21] using the Boolean operators [22]. Searching string included combinations of Medical Subject Heading terms and keywords: (preterm infant OR preterm baby OR premature infant OR premature baby) AND (cultural determinants OR cultural practice OR cultural factors) AND (parents OR mothers OR fathers OR caregivers) AND (neonatal intensive care unit OR NICU OR newborn intensive care unit). The search for unpublished studies (grey literature) used Google Scholar and Google. The integrative review focused on articles that discussed culturally sensitive care when caring for preterm infants. Articles published in English, between 2011 and 2021, met the inclusion criteria for screening. Articles published in the last 10 years or less are recommended for academic reference purposes [23]. There was a consideration of articles published in languages other than English if the abstract was in English. Excluded were studies published before 2011, and all articles addressing the generic care of preterm infants; the comprehensive search yielded 141 articles.

#### Screening

**Fig. 1:**
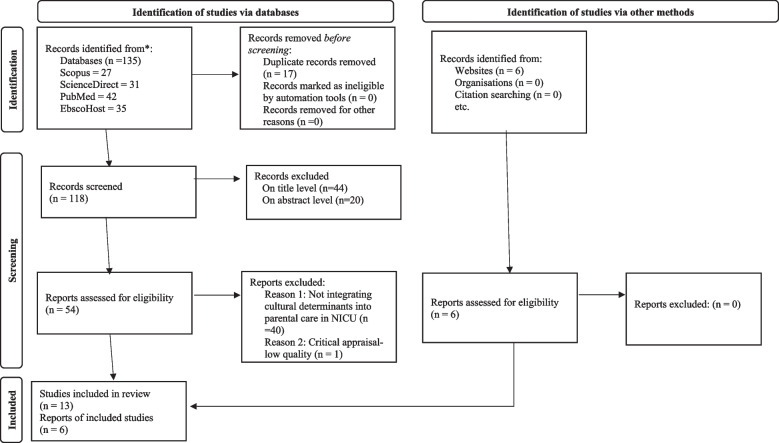
PRISMA 2020 flow diagram (Page MJ, McKenzie JE, Bossuyt PM, Boutron I, Hoffmann TC, Mulrow CD, et al. 2021)

Three reviewers [MN, KDS, SSM] screened the articles to be included in the current review using EPPI Reviewer software, and the promotor [WL] served as the fourth reviewer, assisting in screening articles for which the three reviewers were unable to reach a consensus [23]. All retrieved articles (*N* = 141) were imported into EPPI Reviewer software for storage and screening. Before the screening process, the EPPI Reviewer software automatically identified 17 duplicated studies, which were eliminated. In total, there were 124 articles screened at the title level, and 44 articles excluded based on titles that were not relevant. If the title was unclear or provided an indication that it may address the review question, it was included for abstract screening. The abstracts of 80 included articles were screened to ensure they contained exact information pertinent to the discussion topic [24], and 20 excluded. No articles in non-English languages that matched the inclusion criteria based on the abstract screening were found, therefore, there was no translation required. Finally, the full text of 60 articles underwent screening for compliance with the eligibility criteria [25], and 40 excluded. All reviewers screened the reference lists of the included articles, but no new articles were added to the review. Armed with the content of 20 articles selected, based on full-text level, the reviewers justified the excluded studies. The reviewers documented the screening process using the Preferred Reporting Items for Systematic Reviews and Meta-Analyses (PRISMA) flow diagram (**See** Fig. 1) [26].

### Critical appraisal

Critical appraisal is a systematic process for evaluating and determining the methodological quality of published studies [27]. Three reviewers evaluated the methodological quality of 20 (*n* = 20) identified and included studies at the full-text screening level using a John Hopkins Evidence-based Practice Appraisal tool to ensure transparency [28] and reduce the likelihood of bias [29]. The methodological quality appraisal step used EPPI Reviewer software. The choice of John Hopkins Evidence-based Practice Appraisal tool was because of its capability to assess the methodological quality of diverse study designs. All the disparities were resolved through discussion by the reviewers. Twenty (*n* = 20) articles were critically appraised; 14 (*n* = 14) were of high quality, five (*n* = 5) were of good quality, and one (n = 1) was of low quality. Based on the inclusion of articles rated as good or high quality, 19 (*n* = 19) articles were included as the final sample; one article was excluded based on the low methodological quality rating (see Table 1).

**Table 1:** Critical Appraisal

Included studies	Critical appraisal tool	Quality rating	Evidence level
Abdallah B, Whitford H, Bradbury-Jones C, Jones M. Perceptions and attitudes of parents and healthcare professionals about the option of using infant massage in neonatal intensive care units. Journal of Clinical Nursing. 2020;30 (4):499–507.	John Hopkins Evidence-based Practice	High	III
Abdel Razeq NM, Al-Gamal E. Informing mothers of neonatal death and the need for family-centered bereavement care: a phenomenological qualitative study. Journal for Specialists in Pediatric Nursing. 2021;26 (2):1–12	John Hopkins Evidence-based Practice	High	III
Adama EA, Sundin D, Bayes S. Sociocultural practices affecting the care of preterm infants in the Ghanaian community. Journal of Transcultural Nursing. 2021;32 (5):458–465.	John Hopkins Evidence-based Practice	High	III
Brooks JL, Holdtich-Davis D, Docherty SL, Theodorou CS. Birthing and parenting a premature infant in a cultural context. Qual Health Res. 2016;26 (3):387–398.	John Hopkins Evidence-based Practice	High	III
Candelaria LM, Bressler T, Spatz DL. Breastfeeding guidance for Orthodox Jewish families when newborns require special care and continued hospitalization. The American Journal of Maternal Child Nursing. 2019;44 (2):80–85.	John Hopkins Evidence-based Practice	High	IV
Cartagena D, McGrath JM, Reyna B, Parker LA, McInnis J, Gephart S, Newnam K. Strategies to improve mother’s own milk expression in Black and Hispanic mothers of premature infants. Advances in Neonatal Care. 2021;10	John Hopkins Evidence-based Practice	High	V
Cleveland LM, Horner SD. Taking care of my baby: Mexican American mothers in the neonatal intensive care unit. Issues Compr Pediatr Nurs. 2012;35 (4):163–175.	John Hopkins Evidence-based Practice	High	II
Gill, V.R.; Liley, H.G.; Erdei, C.; Sen, S.; Davidge, R.; Wright, A.L.; Bora, S. Improving the uptake of kangaroo mother care in neonatal units: a narrative review and conceptual framework. *Acta Paediatrica, International Journal of Paediatrics*, **2021**, *110* (5), 1407–1416.	John Hopkins Evidence-based Practice	High	V
Heidari H, Hasanpour M, Fooladi M. The Iranian parents of premature infants in NICU experience stigma of shame. Medicinski Arhiv. 2012;66 (1):35–40.	John Hopkins Evidence-based Practice	High	III
Johnson Rolfes J, Christensen K, Gershan LA. Acceptance of traditional Chinese medicine in the neonatal intensive care unit: a launching point. Global Advances in Health and Medicine. 2020;9:2164956120924644	John Hopkins Evidence-based Practice	Good	II
Kim J. A concept analysis on the use of Yakson in the NICU. J Obstet Gynecol Neonatal Nurs. 2016;45 (6):836–841	John Hopkins Evidence-based Practice	High	V
Mann D. Design, implementation, and early outcome indicators of a new family-integrated neonatal unit. Nurs Women’s Health. 2016;20 (2):158–166.	John Hopkins Evidence-based Practice	Low	III
Mukunya D, Haaland MES, Tumwine JK, Ndeezi G, Namugga O, Tumuhamye J, et al. “We shall count it as a part of kyogero:” acceptability and considerations for scale up of single dose chlorhexidine for umbilical cord care in Central Uganda. BMC Pregnancy & Childbirth. 2018;18 (1):476–476.	John Hopkins Evidence-based Practice	High	III
Ngozi FO, Gbiri CAO, Olawale OA. Child handling cultural practices for neuromotor development in infants in a cohort of African population: a prospective analytical study. Journal of Neonatology and Clinical Pediatrics. 2020;10.	John Hopkins Evidence-based Practice	Good	II
Nitin JB, Unnikrishnan VA, Naik NS, Mahantshetti MD, Mallapur SM, Kotian MN. Infant rearing practices in South India: a longitudinal study. J Family Med Prim Care. 2013;2 (1):37–43.	John Hopkins Evidence-based Practice	High	III
Peng NH, Liu HL, Chen CH, Bachman J. Cultural practices and end-of-life decision making in the neonatal intensive care unit in Taiwan. J Transcult Nurs. 2012;23 (3):320–326.	John Hopkins Evidence-based Practice	High	III
Sarapat P, Fongkaew W, Jintrawet U, Mesukko J, Ray L. Perceptions and practices of parents in caring for their hospitalized preterm infants. Pacific Rim International Journal of Nursing Research. 2017;21 (3):220–233.	John Hopkins Evidence-based Practice	Good	III
Thorley V. Milk siblingship, religious and secular: history, applications, and implications for practice. Women Birth. 2014;27 (4):e16–19.	John Hopkins Evidence-based Practice	Good	V
Upadhyay RP, Singh B, Rai SK, Anand K. Role of cultural beliefs in influencing selected newborn care practices in rural Haryana. Journal of Tropical Pediatrics. 2012;58 (5):406–408.	John Hopkins Evidence-based Practice	Good	III
Wiebe A, Young B. Parent perspectives from a neonatal intensive care unit: a missing piece of the culturally congruent care puzzle. J Transcult Nurs. 2011;22 (1):77–82	John Hopkins Evidence-based Practice	High	III
End.			

### Data extraction, synthesis, and analysis

#### Data extraction

Three reviewers [MN, KDS, SSM] manually extracted data using a standardised and pre-designed extraction tool [30]. The information on the extraction tool included author and year, aim/purpose, the population (sample size and characteristics), study context, methodology, and findings. There was no missing data, hence there were no authors contacted. The presentation of the extracted data was in a tabular format to enable data synthesis and analysis (**see** Table 2).

**Table 2:** Data extraction

Authors	Aim/Purpose	Research design	Population (Sample, Sample size, and setting)	Findings
Abdallah et al., 2021	To explore the cultural, organisational, and contextual factors parents and healthcare professionals perceive about the option of implementing infant massage in the Lebanese context.	Qualitative	Setting: Lebanese context, NICU in Three University Hospitals. Sample: 22 Parents and 38 HCPs	• Understanding massage: Participants across FGs agreed that massage is a cultural and intergenerational practice - parents consider it a physiotherapy or exercise session for the infant.
Abdel-Razeq & Al-Gamal, 2021	To understand the lived experience of mothers surrounding the time of being informed of neonatal deaths in ICUs.	Qualitative	Setting: Two NICUs in Amman, Jordan- (each from private and public hospitals) Sample: 18 mothers of dead preterm infants	• Family deciding on how and when to inform the mother regarding the death of the infant. Mother’s relative delivered the news with a spiritual content introductory speech.
Adama et al., 2021	To explore the influence of sociocultural practices on caring for preterm infants in the Ghanaian community.	Qualitative	Setting: Government hospitals (one tertiary and three district hospitals) in two regions of Ghana—Ashanti (Kumasi) and the Western region (Tarkwa). Sample: 21 mothers, nine fathers, and 12 household members.	• Elderly women taking charge of caring for the preterm infant • Bathing the preterm infant with herbs concoction to stimulate weight gain • Special ankle and wrist bracelet (charms) to protect the infant from evil spirits
Brooks et al., 2016	To explore American Indian mothers’ perceptions of parenting their premature infants over their first year of life in the context of their culture, including the birth and hospitalization experience.	Qualitative	Setting: NICU in two medical centers and two pediatric clinics located in southeastern North Carolina. Sample: 17 American Indian mothers of a preterm infant	• Multi-generational care-family member assist in taking care of the infant. • Balancing traditional and non-traditional medicine depending on the nature of the disease (i.e., for fever, parents used Tylenol, and for oral thrush, elderly person (parent’s aunt) who took a piece of fatback and washed off the salt and rubbed it all around his mouth and that next day that thrush was gone.”
Candelaria et al., 2019	To examine Orthodox Jewish practices related to providing human milk and breastfeeding for a sick newborn to help nurses provide culturally competent care.	Review	Setting and sample: not applicable	• A circumcision is performed on the eighth day after birth, and at this time, a Hebrew name is given to a male child. On the first Saturday after birth, a female child is given her Hebrew name in the synagogue. When an infant is critically ill or is not thought to survive, the infant may not be named because it would be perceived negatively if the infant did not survive. • Pasteurized donor human milk may or may not be acceptable to the Orthodox family (no Jew infant may be wet nursed by a non-Jew mother).
Cartagena et al., 2021	To identify evidence-based strategies that encourage and improve mom’s milk expression during NICU stay in Black and Hispanic mothers of premature infants	Review	Setting: Not applicable Sample: 3 qualitative and seven quantitative studies	• NICU’s breastfeeding support practices sensitive to cultural and racial norms. • Promote family engagement.
Cleveland & Horner, 2012	To gain a better understanding of the NICU experience for Mexican American mothers	Qualitative	Setting: Community and one large university affiliated NICU Sample: 15 Mexican American mothers	• Making the meaningful connection- Gestures such as calling the infant by their name. • No privacy for practice such as praying; mother had to pray in front of other mothers. • Leaving personal items (blankets, Bibles, pictures of family members, and clothing for dressing the baby) was a way of comforting the baby. • Importance of family-centered care.
Gill et al., 2021	To propose a conceptual framework encompassing factors that may affect the initiation and maintenance of Kangaroo Mother Care in neonatal units	Review	Setting: Not applicable Sample: Not applicable	• Elderly’s influence/belief on care/KMC- For example, if grandmothers or mothers-in-law did not appreciate KMC as a beneficial intervention, parents were less likely to utilise it. • Further, in some cultures, when infants are carried on the chest, as opposed to the back, there is a presumption that the infant must be unwell or have an anomaly. • Ghana, bathing the child immediately after birth is commonplace, and skin-to-skin contact is rare in the first 24 hours.
Heidari et al., 2012	To explore experiences of Iranian parents with a hospitalized premature infant in NICU and examine sociocultural factors associated with having a less than perfect infant.	Qualitative	Setting: NICUs located at various hospitals spread across the city of Isfahan. Sample: 6 fathers, seven mothers, five nurses, and three physicians specialized in neonatology.	• Iranian cultural norms allow relatives and family members to get too involved in commonly considered private issues (infant care). While the Iranian sociocultural structure is based on glorifying the perfect form and finding a reason to discard anything less than perfect. Negative remarks and hurtful comments may be unavoidable when relatives see the newborn.
Johnson Rolfes et al., 2020	To assess the potential for engagement of patients, families, and staff in the NICU with traditional Chinese medicine therapies	Qualitative	Setting: Not specified, ethical approval granted by University of Minnesota Institutional Review Board. Sample: 60 NICU staff members, 23 breastfeeding mothers of NICU patients, and five neonates.	• Majority of the participants accepted traditional Chinese medicine (acupuncture), reported it as helpful, and could recommend it to others. Also indicated an interest in receiving it in the future.
Kim, 2016	To clarify the meaning of Yakson, especially its potential use as an early intervention to improve mother-infant interaction and attachment in the NICU and to advance the understanding of a concept unique to one culture with potential application in other cultures.	Review	Setting: Not applicable Sample: 17 published literature and two dictionaries	• Yakson expression used in Korean daily life relies on therapeutic touch and psychological bonding, and Yakson is medicine hands.
Mukunya et al., 2012	To explore the acceptability of single-dose chlorhexidine solution for umbilical cord care among health workers and infant care providers in the districts of Kampala and Mukono in Central Uganda.	Qualitative	Setting: Districts of Kampala and Mukono in Central Uganda. Sample: Community key persons-[mothers (18), health workers (8), traditional birth attendants (2), a father (1) and a grandmother (1)] and four focus group discussions [3 with mothers and 1 with health workers].	• Combining western medicine (chlorhexidine use) with herbal medicine. • Bathing the newborn with herbal medicine. • Influence/ taking charge of care by elders (primarily grandmothers and female elders).
Ngozi et al., 2020	To explore the child-handling cultural practices among infants from an African population.	Mixed method	Setting: Tertiary and secondary health institution in Lagos, Nigeria. Sample: 64 neonates and an unspecified number of mothers	• Child handling cultural practices/exercises (trunk stretching, stretching of lower limbs, soft tissue mobilization, supported sitting, supported standing, and supported walking) demonstrated to expedite and have positive outcome/influence on neuromotor development.
Nitin et al., 2013	To determine the rearing practices among infants in a rural area of south India.	Quantitative	Setting: Kinaye Primary Health Centre (PHC) area, Jawaharlal Nehru Medical College’s field practice area in Belgaum District of Karnataka state. Sample: 194 neonates	• Oil massage was done using coconut oil, and it was given before bathing the infant. • Giving a bath to infants on the delivery day, some after two days but within seven days while others after seven days.
Peng et al., 2012	To describe conditions of decision making for dying infants and cultural effects on the process of infant death in the NICU	Quantitative	Setting: Taichung Veterans General Hospital in Taiwan. Sample: 50 charts of neonatal inpatients who died in the NICU between 2002 and 2008.	• Family prayed for the dying infant • Family hung the good luck charm in the incubator
Sarapat et al., 2017	To better understand Thai parental involvement in caring for hospitalized preterm infants.	Qualitative	Setting: Newborn unit of a regional hospital in Eastern Thailand. Sample: 22 parents (19 mothers and three fathers), two grandmothers, and three nurses caring for a preterm infant.	• Prayed for the child to their spiritual anchor
Thorley, 2014	To set out what is milk siblingship and address misconceptions held about it.	Review	Setting: Not applicable Sample: 15 articles	• Milk kinship is unacceptable in the Islam religion. However, if the donor is known to the mother, milk kinship is allowed, but the marriage of children who fed milk from the same mother is prohibited.
Upadhyay et al., 2012	To document the influence of the cultural beliefs on selected newborn care practices at home in the rural areas of Ballabgarh, Haryana	Quantitative	Setting: North India, 28 Villages in the area of Ballabgarh Haryana under Comprehensive Rural Health Services Project. Sample: 415 mothers	• Most of the mothers had some belief but these can be regarded as neutral practices as in most of the households this was not a deterrent to seek care outside home, in case of neonatal illness
Wiebe & Young, 2011	To explore parent (client/patient) perceptions of culturally congruent care within a tertiary NICU based on interviews with culturally diverse families with hospitalized infants	Qualitative	Setting: 51-bed, Level III NICU, Tertiary hospital located in downtown Edmonton, Alberta. Sample: 21 culturally diverse families with hospitalized infants	• Female family members assisting in caring for the infant/ close involvement of family members • Integration of spirituality into the care and healing (praying for the infant) • Grandmothers and aunts singing traditional songs to the infant
End.				

#### Data synthesis and analysis

Data synthesis is a process that entails the aggregation of specific data from various previous studies to develop new concepts or themes [31]. Braun and Clarke’s framework analysed the extracted data and translated the concepts from the identified studies into the current review, thereby evolving new theoretical perspectives that addressed the review question [32]. The data analysis was repetitive and used a step-by-step strategy. The initial step involved reading the extracted data multiple times to obtain an overall understanding of the data. As demonstrated in the data extraction table (see table 2), statements pertinent to the review questions were identified and underlined. The table matrix was used to document and synthesise the identified statements by facilitating the display of patterns and correlations of extracted data across sources (categorisation) [33–35]. Through a series of comparisons, reviewers established borders between the categories, and named and refined them. The iteration process focused on the extracted data as a whole and pertinent statement until reviewers found and agreed on the mutual final themes.

## Results

Data synthesis and analysis from 19 articles (ten qualitative, three quantitative, five review and one mixed method articles) demonstrated the integration of cultural determinants into the care for parents of a preterm infant in the NICU. The 19 articles included represented 12 countries, each with their own distinct culturally based infant care approach. The 12 countries include Canada, China, Ghana, India, Iran, Jordan, Lebanon, Nigeria, Taiwan, Thailand, the United States of America, and Uganda. Although these countries were represented, the included studies only provided cultural information about a subset of the populations. The main themes identified are spiritual care practices, intragenerational infant-rearing practices, infant physical care practices, and combining treatment practices (see Table 3).

**Table 3:** Themes identified from data synthesis

Theme	Sub-theme	N^**a**^	Studies that informed the themes
Spiritual care practices	Religiously influenced feeding practices	2	Candeleria et al.*,* 2019; Thorley et al.*,* 2014
	Religious observances	5	Abdel-Razeq et al.*,* 2021; Sarapat et al.*,* 2017; Cleveland & Horner 2012, Peng et al.*,* 2012; Wiebe & Young, 2011
	Infant naming	2	Candeleria et al.*,* 2019; Cleveland & Horner, 2012
	Symbols of fortune	3	Adama et al. 2021; Cleveland & Horner 2012; Peng et al., 2012
Intragenerational infant-rearing practices	Senior family members influences	4	Adama et al.*,* 2021; Gill et al.*,* 2020; Mukunya et al., 2018; Wiebe & Young, 2011
	Family involvement	8	Abdel-Razeq et al., 2021; Cartagena et al., 2021; Adama et al.*,* 2020; Abdallah et al.*,* 2020; Brooks et al.*,* 2016; Cleveland & Horner, 2012; Heidari et al.*,* 2012; Peng et al., 2012; Wiebe & Young, 2011
Infant physical care practices	Infant cultural bathing	5	Adama et al., 2021; Gill et al.*,* 2020; Mukunya et al., 2018; Nitin et al., 2013; Peng et al., 2012
	Infant massage	4	Johnson Rolfes et al.*,* 2020; Abdallah et al., 2020; Kim, 2016; Nitin et al., 2013
	Infant cultural handling/positioning	2	Gill et al.*,* 2020; Ngozi et al., 2020
Combining treatment practices	Concurrent use of traditional and western medication	4	Mukunya et al.*,* 2018; Brooks et al.*,* 2016; Upadhyay et al.*,* 2012; Wiebe & Young, 2011
End.			

N^a^ = number of papers

### Spiritual care practices

Spiritual care has developed into a fundamental and significant component of comprehensive healthcare. The guide for spiritual care practices is an individual’s convictions, actions, emotions, and experiences to relate to the divine as a source of hope [36, 37]. For this review, spiritual care practices include religiously influenced feeding practices, religious observances, infant naming, and symbols of fortune.

#### Religiously influenced feeding practices

Two articles included in this review focused on religious considerations as part of the culture when discussing infant feeding practices. The term “feeding practice” refers to milk feeding methods, such as direct breastfeeding, donor milk, and formula feeding. One study stated that Jewish cultural considerations for milk kinship include that only a Jewish mother can be a milk donor to a Jewish preterm infant [34]. According to Islamic culture, a milk donor can be a mother from any culture but known to the parents of the recipient’s preterm infant [38]. Additionally, both studies [38, 39] stated that Jewish and Islamic cultures prohibit marriage between infants who share breast milk due to them considered as “milk kinship siblings”.

#### Religious observances

Parents primarily used prayer to request healing for their preterm infants and drew hope from the spiritual anchor. Five studies conducted in Jordan [40], Thailand [41], Taiwan [42], the United States [43] and Canada [44] found that parents prayed for spiritual protection and healing for their infants and were joined in their prayers by family members and healthcare professionals. Conversely, other studies [42, 43] demonstrated that some parents individually prayed for their infants but lacked the necessary privacy. With regard to dreadful news, such as the death of an infant, family members were the ones who communicated the bad news to parents through an introduction speech with spiritual content, which is regarded as the least painful way to receive difficult news [40].

#### Infant naming

Infant naming can be viewed positively or negatively depending on cultural practices and spiritual beliefs. A study by Candelaria et al. [39] pointed out that Jewish society values infant naming, with a male infant being named on the eighth day after birth and a female infant being given a Hebrew name in the temple on the first Sabbath following birth. In addition, a study by Cleveland et al. [43] revealed that addressing the infant by their first name establishes a meaningful connection between the parents and the infant and healthcare professionals. Conversely, from a different perspective, critically ill infants are not named in Jewish culture because their death would be negatively perceived if they did not survive [39].

#### Symbols of fortune

For centuries, fortune symbols have been integral to various cultural expressions associated with the spiritual belief that they bring good fortune. Studies indicate that fortune symbols were utilised with the belief that they conferred protection and brought luck to the retainer [42, 43, 45]. Although these studies relate to the function of fortune symbols, they all used different lucky items. For instance, in Ghana [45], wrist and neck bracelets are used to keep evil eyes away from the infant, and in Taiwan [42], parents hang a good-luck charm such as a Buddhist Dharma wheel in the incubator for protection against the devil. Additionally, Mexican Americans kept personal items such as blankets, bibles, family photographs, and clothing with the preterm infant, believing that the personal items would comfort the preterm infant and create a connection between the preterm infant and the family [43].

### Intragenerational infant rearing practices

In this review, intragenerational infant rearing practice is a term that may refer to the process of caring for an infant within a family by the family to assist with daily care and influence parental behaviour and attitudes toward the next new parents. The main concepts that constitute intragenerational infant rearing practices are senior family members’ influences and family involvement.

#### Senior family members’ influences

In certain countries, the senior family members maintain a position of prominence within the family structure, retaining power and influence over the family, including infant rearing practices. The authors of the included articles revealed that in countries such as Ghana [45], Uganda [46], and Canada [44], senior family members, particularly elderly females, were involved in caring for preterm infants. For instance, grandmothers were reported to lead the daily care activities for preterm infants, such as bathing after hospital discharge [45, 46].

Furthermore, grandmothers culturally have power and influence over the care of preterm infants, even if in conflict with medical practice [45, 47]. For example, one of the Ghanaian parents reported that “*When one day, I challenged my mother-in-law that she was not supposed to give my baby an enema, she got upset with me and called me ungrateful and disrespectful. I had to allow her to do her job because she has cared for eleven children”* [45].

#### Family involvement

Family involvement offers comprehensive care for parents of preterm infants in relation to cultural practices. Seven studies conducted in Jordan [40], Taiwan [42], Canada [44], the United States [48, 49], Lebanon [50], and Iran [51] indicated that extended family members supplement the preterm infant’s care provided by parents. An example of this is family members assisting the parents with daily preterm infant care [40, 42, 44, 49] and decision-making contributions related to preterm infant care, preserving family values and cultural customs [50]; conversely, Iranian culture promotes family involvement, family members make negative and offensive remarks regarding preterm infants because Iranians do not perceive preterm infants as normal human beings [51].

### Infant physical care practices

Physical care practices reflect the daily values and belief systems of families and their cultures within communities [48]. Infant physical care practices entail preterm infant bathing, infant massage, and infant cultural handling and position.

#### Infant bathing

Infant bathing is one of the cultural infant-rearing techniques practiced in different ways and for various reasons. Studies conducted in Ghana [45] and Uganda [46] reported that infants are bathed with water and traditional herbal concoctions to stimulate weight gain and protection from evil spirits. Additionally, bathing times vary, and it is common for infants to be bathed shortly after delivery in Ghanaian culture [47]. One study conducted in South India revealed that although most mothers bathed their infants on the delivery day, some infants were bathed two to seven days after birth; the authors did not provide specific reasons why the bathing of infants was on different days [52]. Another study found in Taiwan that infants who died in the NICU received a bath shortly after death [42].

#### Infant massage

Infant massage is a cultural practice that can be used to reduce pain and provide physiotherapy benefits. A study conducted in Lebanon [50] and India [52] reported that massage was with various oil-based products and that parents considered it a physiotherapy session for the infant. In the same vein, in China, another method of massaging is with the traditional touch called Yakson, in which parents caress their sick infant with their bare hands in the hope that their hands will alleviate the infant’s pain [53]. Furthermore, massage was reportedly a therapy to relax infants, boost their vagal activity and stomach motility, and eventually improve weight gain [54].

#### Infant cultural handling and positioning

Infant handling and positioning are meaningful on a cultural level. In the Nigerian culture, infant handling techniques, such as stretching infants’ limbs, aid neuro-motor development [55]; for example, the purpose of stretching limbs of infants aged 0–3 months was to ensure joint flexibility, bone strengthening, and alignment [55]. In Columbia, carrying infants on the chest facilitates kangaroo mother care; however, in certain cultures (authors did not specify), carrying infants on the chest rather than the back implies that the infant is ill or abnormal [47].

### Combining treatment practices

Combined treatment practices may refer to the utilisation of traditional medical practices and western medical practices. Combining treatment practices in this review are the concurrent use of traditional and western medications to treat preterm infants.

#### Concurrent use of traditional and western medication

Evidence supports integrating cultural practices into modern treatments when caring for preterm infants. Two studies done in Uganda [46] and the United State [49] indicated that parents were incorporating cultural treatment practices into western medicine depending on the type and nature of the condition of the infant, as an example, some Lumbee parents in the United States would offer over-the-counter drugs for conditions such as fever, but the same parents would take their infant to their aunt to wash off the oral thrush with pieces of fatback and salt, which were effective [49]. Similarly, the Ugandan parents used chlorhexidine, which was better at dealing with foul umbilical cord smell than alternatives; however, the belief was that the use of kyogero, an herbal mixture, hastened umbilical cord separation [46]. Finally, in studies conducted in Canada [44] and India [56], parents indicated they would seek and implement medical treatment for their infant regardless of their beliefs in and use of home remedies.

## Discussion and recommendations

The current review explored and synthesised evidence on integration of cultural determinants into caring for parents of preterm infants in the NICU. The review findings suggest it is possible to integrate cultural determinants, such as spiritual care practices, intragenerational infant-rearing practices, infant physical care practices, and intertwining treatment practices, into care of a preterm infant in the NICU as an intervention to ensure and promote culturally sensitive care.

The findings of this review demonstrate that utilisation of pooled human milk is culturally conditioned. Using human milk in Islamic societies necessitates the identification of the donor by the parents of the recipient to prevent future incest marriage between milk siblings; previous research noted similar findings [57, 58]. The findings contradicted those of the studies conducted in Malaysia [59], South Africa [60], France [61], China [61], and Spain [62]. Another cultural consideration is the prohibition of non-Jewish mothers from wet nursing Jewish infants. In the same vein, previous studies have indicated that Orthodox Jews may request breast milk from kosher-keeping mothers [63, 64]; the rationale could be that kosher-keeping donors follow the same religious dietary beliefs as the mother of the recipient. These conditions need consideration when using pooled human milk in the NICU to feed Islamic and Orthodox Jews preterm infants in a culturally appropriate way.

This review indicates that senior family members have the power to influence infant-rearing practices. The finding is in accordance with the previous evidence in which senior family members, such as grandmothers, were perceived as knowledgeable, experienced, and significant sources of support in infant-rearing practices [65–70]. An explanation for this result could be the cultural norm that the senior family member is typically the primary decision-maker in the family. Despite their influential positions, senior family members preferred cultural care practices rather than approved contemporary healthcare practices [71]. These findings could be an indication that senior family members should be engage in health programmes, such as education and counselling sessions regarding contemporary care practices, to ensure safe, culturally sensitive infant-rearing practices.

Integrating family into caring for a preterm infant in this review demonstrated the value of sustaining family and cultural practices as well as support. Literature suggests that this is a common occurrence [69, 72–74]. Conversely, previous studies established poor paternal support which led to the abdication of overall responsibility for infant care to mothers [75, 76]; this could be because in most cultural settings, fathers do not actively participate in the care of infants. Another explanation could be that certain families and cultures denigrate preterm infants, leading fathers to blame mothers for delivering preterm infants; for instance, a study conducted in Ethiopia described preterm infants using idioms and memes such as “*little rat size and sick child*” [75]. There is a need to discourage this unsupportive practice as it can lead to detrimental health outcomes such as stress, postpartum depression, and loneliness for mothers, consequently affecting the infant.

The practice of infant ritual bathing beliefs was an inheritance from the older generations. The current review found that Ghanaian and Ugandan parents perform the initial infant ritual bath at various times and with herbal medicine in the water. The findings corroborate previous research, which indicated that bathing should begin within six hours of delivery [77–79], within one week [80], and on day 9 or 11 with turmeric [81]. The performing of ritual bathing was for various reasons, including removing sperm and blood, alleviating odour, strengthening the infant, and preventing sickness in the infant [81, 82]. While acknowledging the various cultural bathing times, the recommendation is to delay the first infant bath for a full day [83]; however, if this is not possible for cultural reasons, the bath needs delaying for at least six hours to improve infant-maternal bonding and prevent hypothermia, hypoglycaemia, and dry skin [83]. To address infant bathing beliefs and values, the NICU healthcare professional should enquire with the parents regarding their preference in terms of infant bathing.

The practice of massaging an infant is an old phenomenon. In this review, the massaging of the infant in Lebanon and India was with oil-based products. There were similar findings observed in studies conducted in Pakistan [84], India [85, 86] sand Nepal [87]. Like in China, Yakson therapy as reported in the current review, gentle physical contact plays a significant role in healthy development [88]; additionally, the review indicated that infant massage therapy had beneficial health effects. Consistent with this finding, infant massage has shown to promote motor development [79] and weight gain in preterm infants [89], therefore, healthcare professionals should consider integrating infant massage sessions for preterm infants into the NICUs, guided by the cultural backgrounds of the parents.

In most cultures, it is culturally accepted to use traditional medicine [90, 91]. The review indicates that parents exhibited dualistic responsibility to care for the infant, both culturally and medically. Similar findings emphasise that parents initially used traditional home remedies before seeking medical treatment [69]. On the same note, literature further reported the use of traditional drying agents, such as ashes, soot, and cow dung [92], charcoal, powder, and dust [93] were to facilitate umbilical cord dryness, while chlorhexidine was seen to prolong the separation. In this review, United State Lumbee, Ghanaian, India, and Ugandan parents demonstrated the belief that both medical and traditional medicine contribute equally to the care of infants. While acknowledging cultural practices, harmful practices, such as applying ashes, cow dung, and dust needs to be discouraged as they pose a risk of infection.

Infant naming is rooted in specific rituals and practices and regarded as a significant affair with varying cultural connotations [94]. The review noted that Jewish infants receive names on specific days following birth to commemorate religious observances. Previous research has indicated a strong association between infant naming practice and spirituality [95]. The association could be that the infant names are more indicative of their family ties, the tribe of origin, and religious affiliations. Infant naming can occur a few days after birth [96]; this delay in naming could be due to the consultation of senior family members for advice and approval. Furthermore, the review revealed that Jewish culture prohibits naming of a critically sick infant due to its negative perceptions, particularly if the infant dies. Two studies [97, 98] made similar findings, emphasising that infant naming, even of an alive infant, can result in negative consequences, such as discrimination, bullying, and teasing by friends and peers. Therefore, to integrate culturally based infant naming practice, the care team should ask parents how and when will they like to name their infants.

Religious beliefs are an integral and significant part of a culture. The review found that parents from Jordan, Thailand, Taiwan, the United States and Canada relied on prayer and entrusted God for spiritual comfort and infant healing. The current review findings are consistent with previous research [99–103]. A study conducted in India reported that prayer was the daily religious and spiritual routine geared towards the admitted infant for healing [99]; this suggests parents use religious beliefs or beliefs in God as one of the coping mechanisms during an infant’s stay in the NICU. Therefore, parental religious and spiritual needs should be integrated into preterm infant care to construct a culturally sensitive and informed support structure for parents of preterm infants in the NICU. The NICU healthcare professionals could implement this by informing the religious parents of areas that are designated for prayer within the hospital premises, e.g. hospitals chapel building.

The use of lucky items is practiced in certain cultures. The current review found that in Ghana, religious objects were kept with the infant to prevent misfortune against the infant while Mexican Americans parents used personal belongings as a source of comfort to the infant. Similar findings were reported by two studies which documented that the parents employed traditional applicants for the infants to prevent illness and cast evil spirits; examples of lucky items used include neck, ankle, and wrist bands [102, 104]. Healthcare professionals in NICU settings need to be culturally sensitive when attending to newborns with these lucky items.

The review a study done in Nigeria indicates that infant positioning and handling had health benefits for the infant. Previous studies reported similar findings [105, 106]. Examples include stretching limbs, holding the infant tight on the mother’s chest or back, shushing, swaddling, and lying in a prone position [107, 108]; however, while other culturally accepted positions were beneficial, they also pose a health risk to the infant. Swaddling, for example, has been shown to reduce cold exposure and improve sleep patterns [108, 109], but it is also a significant risk factor for hip dysplasia [110]. Furthermore, carrying infants on the chest is known as the “kangaroo mother care position,” however, the review found that in other cultures not specified by the author, it denotes the condition of the infant, such as being ill or abnormal. There were no previous similar findings identified, which may imply that less is known about chest carrying positions or handling practices in other cultures, which warrants further research.

## Conclusion

The current review provides significant insights regarding the integration of cultural determinants of parents into the care of preterm infants to improve culturally sensitive healthcare services. The findings highlight that most infant-rearing practices are influenced by or based on spiritual care, intergenerational infant-rearing practices, physical care, and combining traditional and western treatment. All cultural practices must be safe for use when providing healthcare services, however, the review revealed that certain cultural practices are not always safe, even though they may be significant to the parents’ cultural beliefs. Such unsafe practices should therefore be discouraged. Consequently, there is a need for parents and families to be educated on safe cultural practices, for healthcare professionals to be educated on the safe integration of cultural determinants of parents into preterm infant care in the NICU, and for healthcare policies to serve as guidelines for culturally sensitive healthcare services that are well-balanced with infant safety. However, a gap exists on how integration of cultural determinants in NICU setting can be implemented. Therefore, further research is recommended to develop a conceptual framework or model on how best can cultural practices be integrated in NICU care approach to ensure comprehensive culturally sensitive care.

## Supplementary Information

**Additional file 1:** **Supplementary Table 1.** Title Screening (EPPI Reconciliation Report)

**Additional file 2:Supplementary Table 2.** Abstract Screening (EPPI Reconciliation report)

**Additional file 3:Supplementary Table 3a.** Full text screening (Manual Reconciliation report)

**Additional file 4:Supplementary Table 4 a and b.** Data matrix with themes

## Data Availability

The dataset used and analysed during the current review are available from the corresponding author on reasonable request.

## Abbreviations

NICU: Neonatal Intensive Care Unit
NWU: North-West University
PRISMA: Preferred Reporting Items for Systematic Reviews and Meta-Analyses.
