# Supplementary material for: Exploring cultural determinants to be integrated into preterm infant care in the neonatal intensive care unit: an integrative literature review
Source: BMC Pregnancy Childbirth. 2023 Jan 9;23:15. doi: 10.1186/s12884-022-05321-7 (PMC9830862; doi:10.1186/s12884-022-05321-7)
Supplement: Supplementary file 1 — Additional file 1: Supplementary Table 1. Title Screening (EPPI Reconciliation Report) [file 12884_2022_5321_MOESM1_ESM.pdf]

Supplementarty Table 1: Title Screening (EPPI Reconciliation Report)

ItemId	ShortTitle	Title	I/E/D/S flag	Include on title	Include for second opinion	Exclude (for any other reason/s)
			FullPath:	Include on title	Include for second opinion	Exclude (for any other reason/s)
62548540	Abdallah (2021)	Perceptions and attitudes of parents and healthcare professionals about the option of using infant massage in neonatal intensive care units	(I) Included	Madimetja Nyaloko **Salaminah Moloko-Phiri**		
62548614	Abdel (2021)	Informing mothers of neonatal death and the need for family-centered bereavement care: A phenomenological qualitative study.	(I) Included	Madimetja Nyaloko **Salaminah Moloko-Phiri**		
62548570	Abstract Book (2013)	Abstract Book	(I) Included			Madimetja Nyaloko **Salaminah Moloko-Phiri**
62548579	Abstracts (2011)	Abstracts	(I) Included			Madimetja Nyaloko **Salaminah Moloko-Phiri**
62548587	Abstracts (2019)	Abstracts	(I) Included			Madimetja Nyaloko **Salaminah Moloko-Phiri**
62815571	Adama et al (2020)	Sociocultural Practices Affecting the Care of Preterm Infants in the Ghanaian Community	(I) Included	**Madimetja Nyaloko** Khumo Shopo		
62548629	Aftyka (2017)	Support provided by nurses to parents of hospitalized children - cultural adaptation and validation of Nurse Parent Support Tool and initial research results.	(I) Included	**Madimetja Nyaloko**		Khumo Shopo
62548575	Altimier (2016)	The Neonatal Integrative Developmental Care Model: Advanced Clinical Applications of the Seven Core Measures for Neuroprotective Family-centered Developmental Care	(I) Included	Madimetja Nyaloko		**Salaminah Moloko-Phiri**
62548582	APPD Abstracts (2021)	APPD Abstracts	(I) Included			Madimetja Nyaloko

						**Salaminah Moloko-Phiri**
62548557	Arnold (2016)	Simulation training for primary caregivers in the neonatal intensive care unit	(I) Included	**Madimetja Nyaloko**		Khumo Shopo
62548592	Ashby (2016)	Infant Mental Health with High Risk Populations	(I) Included			**Madimetja Nyaloko** Khumo Shopo
62548654	Ateşoğlu (2018)	Sociodemographic risk factors for febrile seizures: A school-based study from Izmir, Turkey.	(I) Included			**Madimetja Nyaloko** Khumo Shopo
62548545	Bayih (2020)	The burden of traditional neonatal uvulectomy among admissions to neonatal intensive care units, North Central Ethiopia, 2019: A triangulated crossectional study	(I) Included	**Salaminah Moloko-Phiri**	Madimetja Nyaloko	
62815569	Beinempaka (2014)	The practice of traditional rituals and customs in newborns by mothers in selected villages in southwest Uganda	(I) Included	**Madimetja Nyaloko** Khumo Shopo		
62548583	Berkowitz (2012)	Sudden Infant Death Syndrome, Sudden Unexpected Infant Death, and Apparent Life-Threatening Events	(I) Included			**Madimetja Nyaloko** Khumo Shopo
62548574	Best Papers from the... (2020)	Best Papers from the last 10 years	(I) Included			Madimetja Nyaloko **Salaminah Moloko-Phiri**
62612580	Birthing and Parenting a Premature Infant in a Cultural Context	Birthing and Parenting a Premature Infant in a Cultural Context	(I) Included	**Madimetja Nyaloko** Khumo Shopo		
62548560	Bonet (2015)	Approaches to supporting lactation and breastfeeding for very preterm infants in the NICU: A qualitative study in three European regions	(I) Included	**Madimetja Nyaloko** Khumo Shopo		
62548624	Bracht (2012)	Strategies for reducing the risk of respiratory syncytial virus infection in infants and young children: a Canadian nurses' perspective.	(I) Included			**Madimetja Nyaloko** Khumo Shopo
62548630	Bracht (2012)	Identifying and ensuring optimal care for all children at risk of developing serious respiratory syncytial virus	(I) Included			Madimetja Nyaloko **Salaminah Moloko-Phiri**

		disease: a Canadian nurses' perspective.				
62548632	Bracht (2012)	Practical resources for nurses and other health care providers involved in the care of children at risk for respiratory syncytial virus infection.	(I) Included			**Madimetja Nyaloko** Khumo Shopo
62548651	Breastfeeding Guidance... (Candelaria)	Breastfeeding Guidance for Orthodox Jewish Families When Newborns Require Special Care and Continued Hospitalization.	(I) Included	Khumo Shopo	**Madimetja Nyaloko**	
62548664	Brenneman (2014)	Couplet Care: The Magic Within.	(I) Included		Khumo Shopo	**Madimetja Nyaloko**
62548605	Brockway (2020)	Does breastfeeding self-efficacy theory apply to mothers of moderate and late preterm infants? A qualitative exploration.	(I) Included	Khumo Shopo	**Madimetja Nyaloko**	
62548598	Brødsgaard (2019)	Parents' and nurses' experiences of partnership in neonatal intensive care units: A qualitative review and meta-synthesis.	(I) Included	Madimetja Nyaloko **Salaminah Moloko-Phiri**		
62548628	Brooten (2016)	Death Rituals Reported by White, Black, and Hispanic Parents Following the ICU Death of an Infant or Child.	(I) Included		**Madimetja Nyaloko** Khumo Shopo	
62548666	Bulpitt (2014)	Implementing Use of Donor Breast Milk in the Well Baby Population: It's Not Just for the NICU Any More.	(I) Included		**Madimetja Nyaloko**	Khumo Shopo
62548595	Cardin (2015)	Neuroprotective Core Measures 1–7: A Developmental Care Journey: Transformations in NICU Design and Caregiving Attitudes	(I) Included	Madimetja Nyaloko **Salaminah Moloko-Phiri**		
62548541	Cartagena (2021)	Strategies to Improve Mother's Own Milk Expression in Black and Hispanic Mothers of Premature Infants	(I) Included	Madimetja Nyaloko **Salaminah Moloko-Phiri**		
62548627	Chen (2019)	The Effectiveness of an Intervention Program for Fathers of Hospitalized Preterm Infants on Paternal Support and Attachment 1 Month After Discharge.	(I) Included	**Salaminah Moloko-Phiri**	Madimetja Nyaloko	
62548580	Clarkson (2021)	Former neonatal intensive care unit fathers' involvement 4 years later: A qualitative study	(I) Included	**Salaminah Moloko-Phiri**	Madimetja Nyaloko	
62548612	Cleveland (2012)	Taking care of my baby:	(I)	Madimetja		

		mexican-american mothers in the neonatal intensive care unit.	Included	Nyaloko **Salaminah Moloko-Phiri**		
62548625	Cleveland (2013)	"Try not to judge": mothers of substance exposed infants.	(I) Included		**Salaminah Moloko-Phiri**	Madimetja Nyaloko
62548621	Craighead (2014)	The influence of early-term birth on NICU admission, length of stay, and breastfeeding initiation and duration.	(I) Included	**Salaminah Moloko-Phiri**	Madimetja Nyaloko	
62548620	Cricco-Lizza (2016)	Infant Feeding Beliefs and Day-to-Day Feeding Practices of NICU Nurses.	(I) Included	**Madimetja Nyaloko**	Khumo Shopo	
62548555	Daglas (2018)	Ethical decision making in neonatal intensive care: Adaptation of EURONIC research protocol in Greece	(I) Included		**Madimetja Nyaloko**	Khumo Shopo
62548554	Dall'Oglio (2018)	Neonatal intensive care parent satisfaction: a multicenter study translating and validating the Italian EMPATHIC-N questionnaire	(I) Included	Khumo Shopo	**Madimetja Nyaloko**	
62548585	Dam (2017)	Feeding premature neonates: Kinship and species in translational neonatology	(I) Included	**Madimetja Nyaloko** Khumo Shopo		
62548652	Deng (2018)	Early father-infant skin-to-skin contact and its effect on the neurodevelopmental outcomes of moderately preterm infants in China: study protocol for a randomized controlled trial.	(I) Included	Madimetja Nyaloko **Salaminah Moloko-Phiri**		
62548558	Dykes (2016)	Perceptions of European medical staff on the facilitators and barriers to physical closeness between parents and infants in neonatal units	(I) Included	Madimetja Nyaloko **Salaminah Moloko-Phiri**		
62548576	Edwards (2021)	Following through: Interventions to improve long-term outcomes of preterm infants	(I) Included	Madimetja Nyaloko **Salaminah Moloko-Phiri**		
62548572	Eeles (2020)	Tool to Enhance Relationships Between Staff and Parents in the Neonatal Unit	(I) Included	**Madimetja Nyaloko**	Khumo Shopo	
62548567	Ehrich (2016)	The Child Health Care System of Germany	(I) Included			Madimetja Nyaloko **Salaminah Moloko-Phiri**

62548618	Eom (2019)	The Relationship between Stress, Social Support, and Confidence in Paternal Role Perceived by Korean Fathers of High Risk Infants.	(I) Included	**Madimetja Nyaloko** Khumo Shopo		
62548588	E-Poster Viewing (2019)	E-Poster Viewing	(I) Included			Madimetja Nyaloko **Salaminah Moloko-Phiri**
62548569	Erdei (2021)	Parent mental health and neurodevelopmental outcomes of children hospitalized in the neonatal intensive care unit	(I) Included	**Madimetja Nyaloko** Khumo Shopo		
62548659	Follett (2017)	Implementation of the Neonatal Nurse Practitioner Role in a Community Hospital's Labor, Delivery, and Level 1 Postpartum Unit.	(I) Included	**Salaminah Moloko-Phiri**		Madimetja Nyaloko
62548589	Free Communication (Oral)... (2015)	Free Communication (Oral) Presentations	(I) Included			Madimetja Nyaloko **Salaminah Moloko-Phiri**
62548660	Gagliardi (2017)	Extreme Prematurity Outcomes: Have We Really Reached the Limit?	(I) Included	**Madimetja Nyaloko** Khumo Shopo		
62548581	Ghorbani (2021)	Beliefs and attitudes of nurses towards open visiting policy in neonatal intensive care units: A descriptive cross-sectional study in northwest of Iran	(I) Included			**Madimetja Nyaloko** Khumo Shopo
62548539	Gill (2021)	Improving the uptake of Kangaroo Mother Care in neonatal units: A narrative review and conceptual framework	(I) Included	**Madimetja Nyaloko**	Khumo Shopo	Khumo Shopo
62548657	Govender (2017)	Knowledge and Cultural Beliefs of Mothers Regarding the Risk Factors of Infant Hearing Loss and Awareness of Audiology Services.	(I) Included	**Madimetja Nyaloko**		Khumo Shopo
62548538	Gupta (2021)	Systematic review confirmed the benefits of early skin-to-skin contact but highlighted lack of studies on very and extremely preterm infants	(I) Included	Madimetja Nyaloko **Salaminah Moloko-Phiri**		
62548633	Hariati (2021)	Indonesian mothers' beliefs on caring practices at home	(I) Included	Madimetja Nyaloko		

		for preterm babies after hospital discharge: A qualitative study.		**Salaminah Moloko-Phiri**		
62548672	Heidari (2012)	The Iranian parents of premature infants in NICU experience stigma of shame.	(I) Included	**Madimetja Nyaloko** Khumo Shopo		
62548641	Hidalgo (2020)	Practices following the death of a loved one reported by adults from 14 countries or cultural/ethnic group.	(I) Included			**Madimetja Nyaloko** Khumo Shopo
62548636	Hodges (2015)	Rising above risk: Eliminating infant falls.	(I) Included			**Madimetja Nyaloko** Khumo Shopo
62548548	Holdren (2019)	A qualitative cross-cultural analysis of NICU care culture and infant feeding in Finland and the U.S.	(I) Included	**Madimetja Nyaloko** Khumo Shopo		
62548543	Horbar (2020)	Our Responsibility to Follow Through for NICU Infants and Their Families	(I) Included	**Madimetja Nyaloko** Khumo Shopo		
62548617	Hugill (2013)	Experiences of fathers shortly after the birth of their preterm infants.	(I) Included	Madimetja Nyaloko **Salaminah Moloko-Phiri**		
62548564	Ichijima (2011)	Parental Support in Neonatal Intensive Care Units: A Cross-Cultural Comparison between New Zealand and Japan	(I) Included	**Madimetja Nyaloko** Khumo Shopo		
62548565	Invited Presentations... (2015)	Invited Presentations and Presentations by Organizations and Societies	(I) Included			Madimetja Nyaloko **Salaminah Moloko-Phiri**
62548644	Johnson (2020)	Acceptance of Traditional Chinese Medicine in the Neonatal Intensive Care Unit: A Launching Point.	(I) Included	**Madimetja Nyaloko** Khumo Shopo		
62548661	Jungeun (2017)	Mixed Methods Socio-cultural Study of the Process of Maternal Stress Response in the Neonatal Intensive Care Unit (NICU) in South Korea.	(I) Included	**Madimetja Nyaloko** Khumo Shopo		
62548665	Kennard (2014)	Baby It's Cold Outside.	(I) Included	Khumo Shopo		**Madimetja Nyaloko**
62548551	Khan (2018)	The hearing screening experiences and practices of primary health care nurses: Indications for referral based on high-risk factors	(I) Included			**Madimetja Nyaloko** Khumo Shopo

		and community views about hearing loss				
62548623	Kim (2016)	A Concept Analysis on the Use of Yakson in the NICU.	(I) Included	**Salaminah Moloko-Phiri**	Madimetja Nyaloko	
62548549	Kim (2019)	End-of-Life Care in Neonatal Intensive Care Units from an Asian Perspective: An Integrative Review of the Research Literature	(I) Included	**Madimetja Nyaloko** Khumo Shopo		
62548594	Kim (2020)	Addressing the Needs of Mothers with Infants in the Neonatal Intensive Care Unit: A Qualitative Secondary Analysis	(I) Included	Madimetja Nyaloko **Salaminah Moloko-Phiri**		
62548591	Kondili (2019)	The role of mother-infant bond in neonatal abstinence syndrome (NAS) management	(I) Included	Khumo Shopo	**Madimetja Nyaloko**	
62548604	Kynoe (2020)	When a common language is missing: Nurse-mother communication in the NICU. A qualitative study.	(I) Included	**Madimetja Nyaloko** Khumo Shopo		
62548553	Lantos (2018)	Do sociocultural factors influence periviability counseling and treatment more than science? Lessons from scandinavia	(I) Included			Madimetja Nyaloko **Salaminah Moloko-Phiri**
62548559	Loewy (2015)	NICU music therapy: Song of kin as critical lullaby in research and practice	(I) Included	**Salaminah Moloko-Phiri**		Madimetja Nyaloko
62548619	Mann (2016)	Design, Implementation, and Early Outcome Indicators of a New Family-Integrated Neonatal Unit.	(I) Included	**Madimetja Nyaloko**	Khumo Shopo	
62548635	Marcellus (2016)	Adding to Our Practice Toolkit: Using the ACTS Script to Address Stigmatizing Peer Behaviors in the Context of Maternal Substance Use.	(I) Included			**Madimetja Nyaloko** Khumo Shopo
62548615	Marfurt-Russenberger (2016)	The Experiences of Professionals Regarding Involvement of Parents in Neonatal Pain Management.	(I) Included	Madimetja Nyaloko **Salaminah Moloko-Phiri**		
62548607	Martel (2018)	Nurses' Experiences of End-of-life Photography in NICU Bereavement Support.	(I) Included	Khumo Shopo	**Madimetja Nyaloko**	
62548606	Mefford (2011)	Evaluating nurse staffing patterns and neonatal intensive care unit outcomes using Levine's Conservation Model of Nursing.	(I) Included	**Salaminah Moloko-Phiri**		Madimetja Nyaloko **Salaminah Moloko-Phiri**

62548563	Meier (2013)	Supporting Breastfeeding in the Neonatal Intensive Care Unit. Rush Mother's Milk Club as a Case Study of Evidence-Based Care	(I) Included	**Madimetja Nyaloko** Khumo Shopo		
62548578	Mendes (2017)	Ethical Considerations in Perinatal Palliative Care	(I) Included	**Salaminah Moloko-Phiri**		Madimetja Nyaloko
62548568	Messmer (2014)	ANF Scholars (1955–2012): Stepping Stones to a Nursing Research Career	(I) Included			**Madimetja Nyaloko** Khumo Shopo
62548590	Mirlashari (2019)	Nurses' and Physicians' Experiences of the NIDCAP Model Implementation in Neonatal Intensive Care Units in Iran	(I) Included	**Salaminah Moloko-Phiri**		Madimetja Nyaloko
62548646	Mnisi (2019)	Postnatal depressive features in mothers of neonates admitted to a neonatal unit at Steve Biko Academic Hospital: The role of sociodemographic and psychosocial factors.	(I) Included	Khumo Shopo	**Madimetja Nyaloko**	
62548601	Mohammadi (2020)	Male nursing students' perception of dignity in neonatal intensive care units.	(I) Included	Khumo Shopo		**Madimetja Nyaloko**
62548653	Mukunya (2018)	"We shall count it as a part of kyogero": acceptability and considerations for scale up of single dose chlorhexidine for umbilical cord care in Central Uganda.	(I) Included	**Salaminah Moloko-Phiri**	Madimetja Nyaloko	
62612581	Ngozi (2020)	Child Handling Cultural Practices for Neuromotor Development in Infants in a Cohort of African Population: A Prospective Analytical Study	(I) Included	Madimetja Nyaloko **Salaminah Moloko-Phiri**		
62815570	Nitin et al (2013)	Infant Rearing Practices in South India: A Longitudinal Study	(I) Included	Madimetja Nyaloko **Salaminah Moloko-Phiri**		
62548655	NOVOROĐENAČKA RAZVOJNA... (SIPL)	NOVOROĐENAČKA RAZVOJNA NJEGA I NADZOR.	(I) Included	**Salaminah Moloko-Phiri**	Madimetja Nyaloko	
62548573	Ogata (2017)	Chapter 14 - Nutrition for Children With Special Health Care Needs	(I) Included			**Madimetja Nyaloko** Khumo Shopo
62548622	Parker (2013)	10 years after baby-friendly designation: breastfeeding rates continue to increase in	(I) Included	**Salaminah Moloko-Phiri**	Madimetja Nyaloko	

		a US neonatal intensive care unit.				
62548577	Paul (2018)	6 - Assessment and intervention in the prelinguistic period	(I) Included			Madimetja Nyaloko **Salaminah Moloko-Phiri**
62548608	Peng (2012)	Cultural practices and end-of-life decision making in the neonatal intensive care unit in Taiwan.	(I) Included	**Madimetja Nyaloko** Khumo Shopo		
62548658	Perceptions and Practices... (Sarapat)	Perceptions and Practices of Parents in Caring for their Hospitalized Preterm Infants.	(I) Included	**Madimetja Nyaloko** Khumo Shopo		
62548656	Pham (2018)	Factors associated with postpartum depression in women from low socioeconomic level in Argentina: A hierarchical model approach.	(I) Included			**Madimetja Nyaloko** Khumo Shopo
62548571	Poster... (2015)	Poster Presentations	(I) Included			Madimetja Nyaloko **Salaminah Moloko-Phiri**
62548596	Roque (2017)	Scoping Review of the Mental Health of Parents of Infants in the NICU.	(I) Included		Madimetja Nyaloko **Salaminah Moloko-Phiri**	
62548600	Rosenthal (2013)	A meta-ethnography and theory of parental ethical decision making in the neonatal intensive care unit.	(I) Included	Madimetja Nyaloko **Salaminah Moloko-Phiri**		
62548631	Sables-Baus (2012)	An exemplar for evidence-based nursing practice using the Magnet(®) model as the framework for change: oral feeding practice in the neonatal intensive care unit.	(I) Included	Madimetja Nyaloko **Salaminah Moloko-Phiri**		
62548556	Saigal (2016)	In their own words: Life at adulthood after very premature birth	(I) Included			Madimetja Nyaloko **Salaminah Moloko-Phiri**
62548663	Salam (2015)	Effect of emollient therapy on clinical outcomes in preterm neonates in Pakistan: a randomised controlled trial.	(I) Included	**Salaminah Moloko-Phiri**		Madimetja Nyaloko
62548542	Salmani (2020)	Adaptation of the parent readiness for hospital discharge scale with mothers of preterm infants	(I) Included	**Madimetja Nyaloko** Khumo Shopo		

		discharged from the neonatal intensive care unit				
62548584	Schlittenhart (2011)	Preparing Parents for NICU Discharge: An Evidence-Based Teaching Tool	(I) Included	**Madimetja Nyaloko** Khumo Shopo		
62548597	Setiawan (2019)	Understanding the Effects of Neonatal Early Discharge on Parents: A Literature Review.	(I) Included	**Salaminah Moloko-Phiri**	Madimetja Nyaloko	
62548566	Shorey (2016)	Skin-to-skin contact by fathers and the impact on infant and paternal outcomes: an integrative review	(I) Included	Madimetja Nyaloko **Salaminah Moloko-Phiri**		
62548602	Skene (2019)	Developing family-centred care in a neonatal intensive care unit: An action research study.	(I) Included	Madimetja Nyaloko **Salaminah Moloko-Phiri**		
62548647	Spierson (2019)	Professionals' Practices and Views regarding Neonatal Postmortem: Can We Improve Consent Rates by Improving Training?	(I) Included			Madimetja Nyaloko **Salaminah Moloko-Phiri**
62548638	Suhana (2021)	Postpartum depression among Neonatal Intensive Care Unit mothers and its relation to postpartum dietary intake: A review.	(I) Included		Madimetja Nyaloko **Salaminah Moloko-Phiri**	
62548586	Symposium (2019)	Symposium	(I) Included			**Madimetja Nyaloko** Khumo Shopo
62548645	Tahiru (2020)	Exclusive Breastfeeding and Associated Factors among Mothers with Twins in the Tamale Metropolis.	(I) Included	**Madimetja Nyaloko**		Khumo Shopo
62548609	Thorley (2014)	Milk siblingship, religious and secular: History, applications, and implications for practice.	(I) Included	Madimetja Nyaloko		**Salaminah Moloko-Phiri**
62548613	Turner (2014)	The neonatal nurses' view of their role in emotional support of parents and its complexities.	(I) Included	**Madimetja Nyaloko** Khumo Shopo		
62548593	Umberger (2018)	Enhancing NICU parent engagement and empowerment	(I) Included	Madimetja Nyaloko **Salaminah Moloko-Phiri**		
62548670	Upadhyay (2012)	Role of Cultural Beliefs in Influencing Selected Newborn Care Practices in Rural Haryana.	(I) Included	**Madimetja Nyaloko** Khumo Shopo		

62548561	Van McCrary (2014)	A delicate subject: The impact of cultural factors on neonatal and perinatal decision making	(I) Included	**Madimetja Nyaloko** Khumo Shopo		
62548637	Véras (2011)	The Kangaroo Program at a Brazilian maternity hospital: the preterm/low-weight babies' health-care under examination.	(I) Included	**Madimetja Nyaloko** Khumo Shopo		
62548634	Vickers (2015)	Pasteurized Donor Human Milk Maintains Microbiological Purity for 4 Days at 4°C.	(I) Included			**Madimetja Nyaloko** Khumo Shopo
62548616	Wiebe (2011)	Parent perspectives from a neonatal intensive care unit: a missing piece of the culturally congruent care puzzle.	(I) Included	Madimetja Nyaloko **Salaminah Moloko-Phiri**		
62548626	Wooldridge (2021)	Pediatric and neonatal tracheostomy caregiver education with phased simulation to increase competency and enhance coping.	(I) Included			Madimetja Nyaloko **Salaminah Moloko-Phiri**
62548552	Xu (2018)	Childbirth and Early Newborn Care practices in 4 provinces in China: A comparison with WHO recommendations	(I) Included	**Madimetja Nyaloko**	Khumo Shopo	
62548546	Yotani (2020)	Withholding and withdrawal of life-sustaining treatments for neonate in Japan: Are hospital practices associated with physicians' beliefs, practices, or perceived barriers?	(I) Included	Madimetja Nyaloko **Salaminah Moloko-Phiri**	**Salaminah Moloko-Phiri**	
62548611	Youngblut (2013)	Parents' report of child's response to sibling's death in a neonatal or pediatric intensive care unit.	(I) Included	**Salaminah Moloko-Phiri**		Madimetja Nyaloko **Salaminah Moloko-Phiri**
62548544	Yue (2020)	Barriers and facilitators of kangaroo mother care adoption in five Chinese hospitals: A qualitative study	(I) Included	Madimetja Nyaloko	**Salaminah Moloko-Phiri**	
62548562	Zamanzadeh (2013)	Mothers' experiences of infants discharge in Iranian NICU culture: A qualitative study	(I) Included	**Madimetja Nyaloko** Khumo Shopo		

: Articles reviewed by 3rd Reviewer (Resolutions)
