# Supplementary material for: Exploring cultural determinants to be integrated into preterm infant care in the neonatal intensive care unit: an integrative literature review
Source: BMC Pregnancy Childbirth. 2023 Jan 9;23:15. doi: 10.1186/s12884-022-05321-7 (PMC9830862; doi:10.1186/s12884-022-05321-7)
Supplement: Supplementary file 2 — Additional file 2:Supplementary Table 2. Abstract Screening (EPPI Reconciliation report) [file 12884_2022_5321_MOESM2_ESM.pdf]

Comparison report between: *Madimetja Nyaloko* and *Khumo Shopo* and *Salaminah Moloko-Phiri*

This report is based on the status of the database at the time the comparison was created. Any coding **completed** after the comparison was created will be displayed also in the Agreed column.

Screen on Abstract

Supplementary Table 2: Abstract Screening (EPPI Reconciliation report)

Id	Item	Madimetja Nyaloko	Khumo Shopo	Salaminah Moloko-Phiri	**Agreed version**
62548538	Systematic review confirmed the benefits of early skin-to-skin contact but highlighted lack of studies on very and extremely preterm infants	Include on abstract	Exclude -target group	Include on abstract	Include
62548539	Improving the uptake of Kangaroo Mother Care in neonatal units: A narrative review and conceptual framework	Include for second opinion	Exclude-intervention	Include on abstract	Include
62548540	Perceptions and attitudes of parents and healthcare professionals about the option of using infant massage in neonatal intensive care units	Include on abstract		Include on abstract	Include
62548541	Strategies to Improve Mother's Own Milk Expression in Black and Hispanic Mothers of Premature Infants	Include on abstract	Include on abstract		Include
62548542	Adaptation of the parent readiness for hospital discharge scale with mothers of preterm infants discharged from the neonatal intensive care unit	Exclude on evidence	Include for second opinion	Exclude on evidence	Exclude
62548543	Our Responsibility to Follow Through for NICU Infants and Their Families	Exclude on evidence	Exclude on evidence *No abstract for the article and it does not meet eligibility criteria of original research.*		Exclude
62548544	Barriers and facilitators of kangaroo mother care adoption in five Chinese hospitals: A qualitative study	Include on abstract	Include on abstract	Include on abstract	Include
62548545	The burden of traditional neonatal uvulectomy among admissions to neonatal intensive care units, North Central Ethiopia, 2019: A triangulated crossectional study	Include for second opinion		Exclude-intervention	
62548546	Withholding and withdrawal of life-sustaining treatments for neonate in Japan: Are hospital practices associated with physicians' beliefs, practices, or perceived barriers?	Exclude on evidence	Include for second opinion		
62548548	A qualitative cross-cultural analysis of NICU care culture and infant feeding in Finland and the U.S.	Include on abstract		Include on abstract	Include

62548549	End-of-Life Care in Neonatal Intensive Care Units from an Asian Perspective: An Integrative Review of the Research Literature	Include on abstract	Include for second opinion		Include	
62548552	Childbirth and Early Newborn Care practices in 4 provinces in China: A comparison with WHO recommendations	Include on abstract	Exclude -target group			✓
62548554	Neonatal intensive care parent satisfaction: a multicenter study translating and validating the Italian EMPATHIC-N questionnaire	Exclude on evidence	Exclude-intervention	Include for second opinion		✓
62548558	Perceptions of European medical staff on the facilitators and barriers to physical closeness between parents and infants in neonatal units	Include on abstract	Include for second opinion	Include on abstract	Include	
62548559	NICU music therapy: Song of kin as critical lullaby in research and practice	Include on abstract	Include on abstract		Include	
62548560	Approaches to supporting lactation and breastfeeding for very preterm infants in the NICU: A qualitative study in three European regions	Include for second opinion	Exclude-intervention	Include on abstract	Include	
62548561	A delicate subject: The impact of cultural factors on neonatal and perinatal decision making	Include for second opinion	Include on abstract		Include	
62548562	Mothers' experiences of infants discharge in Iranian NICU culture: A qualitative study	Include on abstract		Include on abstract	Include	
62548563	Supporting Breastfeeding in the Neonatal Intensive Care Unit. Rush Mother's Milk Club as a Case Study of Evidence-Based Care	Exclude on evidence	Exclude -target group<br><br>Exclude-intervention		Exclude	
62548564	Parental Support in Neonatal Intensive Care Units: A Cross-Cultural Comparison between New Zealand and Japan	Include on abstract	Include for second opinion	Include on abstract	Include	
62548566	Skin-to-skin contact by fathers and the impact on infant and paternal outcomes: an integrative review	Include on abstract		Include on abstract	Include	
62548569	Parent mental health and neurodevelopmental outcomes of children hospitalized in the neonatal intensive care unit	Exclude-intervention		Include on abstract		✓
62548572	Tool to Enhance Relationships Between Staff and Parents in the Neonatal Unit	Include for second opinion	Include for second opinion	Include on abstract	Include	
62548575	The Neonatal Integrative Developmental Care Model: Advanced Clinical Applications of the Seven Core Measures for Neuroprotective Family-centered Developmental Care	Include on abstract		Include on abstract	Include	
62548576	Following through: Interventions to improve long-term outcomes of preterm infants	Include for second opinion	Exclude -target group			

62548578	Ethical Considerations in Perinatal Palliative Care	Exclude on evidence	Exclude -target group	Exclude on evidence	Exclude
62548580	Former neonatal intensive care unit fathers' involvement 4 years later: A qualitative study	Exclude on evidence		Include on abstract	
62548584	Preparing Parents for NICU Discharge: An Evidence-Based Teaching Tool	Exclude on evidence	Exclude-intervention	Include for second opinion	Exclude
62548585	Feeding premature neonates: Kinship and species in translational neonatology	Exclude on evidence		Include for second opinion	
62548590	Nurses' and Physicians' Experiences of the NIDCAP Model Implementation in Neonatal Intensive Care Units in Iran	Exclude on evidence	Include on abstract		
62548591	The role of mother-infant bond in neonatal abstinence syndrome (NAS) management	Exclude on evidence	Exclude-intervention		Exclude
62548593	Enhancing NICU parent engagement and empowerment	Include for second opinion	Include on abstract		Include
62548594	Addressing the Needs of Mothers with Infants in the Neonatal Intensive Care Unit: A Qualitative Secondary Analysis	Include on abstract	Include on abstract		Include
62548595	Neuroprotective Core Measures 1–7: A Developmental Care Journey: Transformations in NICU Design and Caregiving Attitudes	Include for second opinion		Include for second opinion	Include
62548597	Understanding the Effects of Neonatal Early Discharge on Parents: A Literature Review.	Exclude on evidence	Include on abstract	Include on abstract	Include
62548598	Parents' and nurses' experiences of partnership in neonatal intensive care units: A qualitative review and meta-synthesis.	Include on abstract	Include on abstract	Include on abstract	Include
62548600	A meta-ethnography and theory of parental ethical decision making in the neonatal intensive care unit.	Include on abstract		Include on abstract	Include
62548601	Male nursing students' perception of dignity in neonatal intensive care units.	Exclude on evidence	Exclude -target group		Exclude
62548602	Developing family-centred care in a neonatal intensive care unit: An action research study.	Include on abstract		Include on abstract	Include
62548604	When a common language is missing: Nurse-mother communication in the NICU. A qualitative study.	Include for second opinion		Include on abstract	Include
62548605	Does breastfeeding self-efficacy theory apply to mothers of moderate and late preterm infants? A qualitative exploration.	Include on abstract	Exclude on evidence		
62548606	Evaluating nurse staffing patterns and neonatal intensive care unit outcomes using Levine's Conservation Model of Nursing.	Exclude - target group	Exclude-intervention	Exclude on evidence	Exclude

62548607	Nurses' Experiences of End-of-life Photography in NICU Bereavement Support.	Exclude on evidence	Exclude-intervention	Exclude on evidence	Exclude
62548608	Cultural practices and end-of-life decision making in the neonatal intensive care unit in Taiwan.	Include on abstract	Include on abstract		Include
62548609	Milk siblingship, religious and secular: History, applications, and implications for practice.	Include on abstract		Exclude-intervention	
62548612	Taking care of my baby: mexican-american mothers in the neonatal intensive care unit.	Exclude on evidence	Include on abstract		
62548613	The neonatal nurses' view of their role in emotional support of parents and its complexities.	Exclude - target group	Include on abstract		
62548614	Informing mothers of neonatal death and the need for family-centered bereavement care: A phenomenological qualitative study.	Include on abstract	Include on abstract		Include
62548615	The Experiences of Professionals Regarding Involvement of Parents in Neonatal Pain Management.	Exclude on evidence	Include for second opinion		
62548616	Parent perspectives from a neonatal intensive care unit: a missing piece of the culturally congruent care puzzle.	Include for second opinion	Include for second opinion	Include on abstract	Include
62548617	Experiences of fathers shortly after the birth of their preterm infants.	Include for second opinion	Include on abstract<br>Include for second opinion	Include on abstract	Include
62548618	The Relationship between Stress, Social Support, and Confidence in Paternal Role Perceived by Korean Fathers of High Risk Infants.	Exclude on evidence		Include on abstract	
62548619	Design, Implementation, and Early Outcome Indicators of a New Family-Integrated Neonatal Unit.	Include for second opinion		Include on abstract	Include
62548620	Infant Feeding Beliefs and Day-to-Day Feeding Practices of NICU Nurses.	Exclude on evidence	Exclude -target group	Include on abstract	Exclude
62548621	The influence of early-term birth on NICU admission, length of stay, and breastfeeding initiation and duration.	Exclude on evidence		Include on abstract	
62548622	10 years after baby-friendly designation: breastfeeding rates continue to increase in a US neonatal intensive care unit.	Exclude on evidence		Include on abstract	
62548623	A Concept Analysis on the Use of Yakson in the NICU.	Include on abstract		Include on abstract	Include
62548627	The Effectiveness of an Intervention Program for Fathers of Hospitalized Preterm Infants on Paternal Support and Attachment 1 Month After Discharge.	Exclude-intervention	Include on abstract		
62548628	Death Rituals Reported by White, Black,	Include on	Include on	Exclude on	

	and Hispanic Parents Following the ICU Death of an Infant or Child.	abstract	abstract	evidence	Include
62548629	Support provided by nurses to parents of hospitalized children - cultural adaptation and validation of Nurse Parent Support Tool and initial research results.	Include on abstract		Include on abstract	Include
62548631	An exemplar for evidence-based nursing practice using the Magnet(®) model as the framework for change: oral feeding practice in the neonatal intensive care unit.	Include for second opinion	Include for second opinion		Include
62548633	Indonesian mothers' beliefs on caring practices at home for preterm babies after hospital discharge: A qualitative study.	Include for second opinion	Include on abstract		Include
62548637	The Kangaroo Program at a Brazilian maternity hospital: the preterm/low-weight babies' health-care under examination.	Include for second opinion	Exclude-intervention		
62548638	Postpartum depression among Neonatal Intensive Care Unit mothers and its relation to postpartum dietary intake: A review.	Exclude-intervention		Exclude on evidence	Exclude
62548644	Acceptance of Traditional Chinese Medicine in the Neonatal Intensive Care Unit: A Launching Point.	Include on abstract	Include for second opinion	Include on abstract	Include
62548645	Exclusive Breastfeeding and Associated Factors among Mothers with Twins in the Tamale Metropolis.	Exclude on evidence		Exclude on evidence	Exclude
62548646	Postnatal depressive features in mothers of neonates admitted to a neonatal unit at Steve Biko Academic Hospital: The role of sociodemographic and psychosocial factors.	Exclude on evidence	Include for second opinion	Include for second opinion	Include
62548651	Breastfeeding Guidance for Orthodox Jewish Families When Newborns Require Special Care and Continued Hospitalization.	Include on abstract		Include for second opinion	Include
62548652	Early father-infant skin-to-skin contact and its effect on the neurodevelopmental outcomes of moderately preterm infants in China: study protocol for a randomized controlled trial.	Include on abstract	Include for second opinion	Include on abstract	Include
62548653	"We shall count it as a part of kyogero": acceptability and considerations for scale up of single dose chlorhexidine for umbilical cord care in Central Uganda.	Include on abstract		Include for second opinion	Include
62548655	NOVOROĐENAČKA RAZVOJNA NJEGA I NADZOR.	Include on abstract		Include on abstract	Include
62548657	Knowledge and Cultural Beliefs of Mothers Regarding the Risk Factors of Infant Hearing Loss and Awareness of Audiology Services.	Include for second opinion		Exclude on evidence	
62548658	Perceptions and Practices of Parents in	Include on	Include on	Include on	

	Caring for their Hospitalized Preterm Infants.	abstract	abstract	abstract	Include
62548659	Implementation of the Neonatal Nurse Practitioner Role in a Community Hospital's Labor, Delivery, and Level 1 Postpartum Unit.	Exclude on evidence	Exclude -target group	Exclude on evidence	Exclude
62548660	Extreme Prematurity Outcomes: Have We Really Reached the Limit?	Include for second opinion		Exclude-intervention	✓
62548661	Mixed Methods Socio-cultural Study of the Process of Maternal Stress Response in the Neonatal Intensive Care Unit (NICU) in South Korea.	Include on abstract	Include on abstract		Include
62548663	Effect of emollient therapy on clinical outcomes in preterm neonates in Pakistan: a randomised controlled trial.	Include on abstract	Include on abstract		Include
62548664	Couplet Care: The Magic Within.	Exclude on evidence	Exclude -target group	Include on abstract	Exclude
62548665	Baby It's Cold Outside.	Exclude on evidence	Exclude -target group	Include for second opinion	Exclude
62548666	Implementing Use of Donor Breast Milk in the Well Baby Population: It's Not Just for the NICU Any More.	Exclude on evidence	Exclude -target group	Exclude on evidence	Exclude
62548670	Role of Cultural Beliefs in Influencing Selected Newborn Care Practices in Rural Haryana.	Include for second opinion	Include on abstract		Include
62548672	The Iranian parents of premature infants in NICU experience stigma of shame.	Include on abstract		Include for second opinion	Include
62612580	Birthing and Parenting a Premature Infant in a Cultural Context	Include on abstract	Include on abstract		Include
62612581	Child Handling Cultural Practices for Neuromotor Development in Infants in a Cohort of African Population: A Prospective Analytical Study	Include on abstract		Include on abstract	Include
62815569	The practice of traditional rituals and customs in newborns by mothers in selected villages in southwest Uganda	Include for second opinion	Include for second opinion *NICU and preterm babies not included.*	Exclude on evidence	Include
62815570	Infant Rearing Practices in South India: A Longitudinal Study	Include on abstract	Include on abstract		Include
62815571	Sociocultural Practices Affecting the Care of Preterm Infants in the Ghanaian Community	Include on abstract	Include on abstract	Include on abstract	Include

✓ : Articles reviewed by 3rd Reviewer (Resolutions)
