# Supplementary material for: Exploring cultural determinants to be integrated into preterm infant care in the neonatal intensive care unit: an integrative literature review
Source: BMC Pregnancy Childbirth. 2023 Jan 9;23:15. doi: 10.1186/s12884-022-05321-7 (PMC9830862; doi:10.1186/s12884-022-05321-7)
Supplement: Supplementary file 3 — Additional file 3:Supplementary Table 3a. Full text screening (Manual Reconciliation report) [file 12884_2022_5321_MOESM3_ESM.pdf]

Supplementary Table 3: Full-text Screening (Manual Reconciliation report)

Item ID	Short Title	Title	Year	New Column
62548540	Include ✓ Abdallah (2021)	Perceptions and attitudes of parents and healthcare professionals about the option of using infant massage in neonatal intensive care units/td>	Include 2021 ✓	
62548614	Include ✓ Abdel (2021)	Informing mothers of neonatal death and the need for family-centered bereavement care: A phenomenological qualitative study./td>	Include 2021 ✓	
62815571	Include ✓ Adama et al (2020)	Sociocultural Practices Affecting the Care of Preterm Infants in the Ghanaian Community/td>	2020 Include ✓	
62548629	Exclude Aftyka (2017)	Support provided by nurses to parents of hospitalized children - cultural adaptation and validation of Nurse Parent Support Tool and initial research results./td>	2017 Exclude	
62548575	Exclude Altimier (2016)	The Neonatal Integrative Developmental Care Model: Advanced Clinical Applications of the Seven Core Measures for Neuroprotective Family-centered Developmental Care/td>	Exclude 2016	
62815569	Exclude Beinempaka (2014)	The practice of traditional rituals and customs in newborns by mothers in selected villages in southwest Uganda/td>	Exclude 2014	
62612580	Birthing and Parenting a Premature Infant in a Cultural Context Include ✓	Birthing and Parenting a Premature Infant in a Cultural Context/td>	2016 Include ✓	
62548560	Exclude Bonet (2015)	Approaches to supporting lactation and breastfeeding for very preterm infants in the NICU: A qualitative study in three European regions/td>	2015 Exclude	
62548651	Include ✓ Breastfeeding Guidance... (Candelaria)	Breastfeeding Guidance for Orthodox Jewish Families When Newborns Require Special Care and Continued Hospitalization./td>	Include ✓	
62548598	Exclude Brødsgaard (2019)	Parents' and nurses' experiences of partnership in neonatal intensive care units: A qualitative review and meta-synthesis./td>	Exclude 2019	
62548628	Exclude Brooten (2016)	Death Rituals Reported by White, Black, and Hispanic Parents Following the ICU Death of an Infant or Child./td>	2016 Exclude	
62548595	Exclude Cardin (2015)	Neuroprotective Core Measures 1–7: A Developmental Care Journey: Transformations in NICU Design and Caregiving Attitudes/td>	2015 Exclude	
62548541	Include ✓ Cartagena (2021)	Strategies to Improve Mother's Own Milk Expression in Black and Hispanic Mothers of Premature Infants/td>	2021 Include ✓	
62548627	Exclude Chen (2019)	The Effectiveness of an Intervention Program for Fathers of Hospitalized Preterm Infants on Paternal Support and Attachment 1 Month After Discharge./td>	2019 Exclude	
62548580	Exclude Clarkson (2021)	Former neonatal intensive care unit fathers' involvement 4 years later: A qualitative study/td>	2021 Exclude	
62548612	Include ✓ Cleveland (2012)	Taking care of my baby: mexican-american mothers in the neonatal intensive care unit./td>	2012 Include ✓	

62548652	Exclude Deng (2018)	Early father-infant skin-to-skin contact and its effect on the neurodevelopmental outcomes of moderately preterm infants in China: study protocol for a randomized controlled trial./td>	2018 Exclude	
62548558	Exclude Dykes (2016)	Perceptions of European medical staff on the facilitators and barriers to physical closeness between parents and infants in neonatal units/td>	2016 Exclude	
62548572	Exclude Eeles (2020)	Tool to Enhance Relationships Between Staff and Parents in the Neonatal Unit/td>	2020 Exclude	
62548618	Exclude Eom (2019)	The Relationship between Stress, Social Support, and Confidence in Paternal Role Perceived by Korean Fathers of High Risk Infants./td>	2019 Exclude	
62548569	Exclude Erdei (2021)	Parent mental health and neurodevelopmental outcomes of children hospitalized in the neonatal intensive care unit/td>	2021 Exclude	
62548539	Include ✓ Gill (2021)	Improving the uptake of Kangaroo Mother Care in neonatal units: A narrative review and conceptual framework/td>	2021 Include	✓
62548538	Exclude Gupta (2021)	Systematic review confirmed the benefits of early skin-to-skin contact but highlighted lack of studies on very and extremely preterm infants/td>	2021 Exclude	
62548633	Exclude Hariati (2021)	Indonesian mothers' beliefs on caring practices at home for preterm babies after hospital discharge: A qualitative study./td>	Exclude 2021	
62548672	Include ✓ Heidari (2012)	The Iranian parents of premature infants in NICU experience stigma of shame./td>	2012 Include	✓
62548548	Exclude Holdren (2019)	A qualitative cross-cultural analysis of NICU care culture and infant feeding in Finland and the U.S./td>	2019 Exclude	
62548617	Hugill (2013) Exclude	Experiences of fathers shortly after the birth of their preterm infants./td>	2013 Exclude	
62548564	Ichijima (2011) Exclude	Parental Support in Neonatal Intensive Care Units: A Cross-Cultural Comparison between New Zealand and Japan/td>	2011 Exclude	
62548644	Include Johnson (2020) ✓	Acceptance of Traditional Chinese Medicine in the Neonatal Intensive Care Unit: A Launching Point./td>	2020 Include	✓
62548661	Exclude Jungeun (2017)	Mixed Methods Socio-cultural Study of the Process of Maternal Stress Response in the Neonatal Intensive Care Unit (NICU) in South Korea./td>	2017 Exclude	
62548623	Kim (2016) Include ✓	A Concept Analysis on the Use of Yakson in the NICU./td>	2016 Include	✓
62548549	Exclude Kim (2019)	End-of-Life Care in Neonatal Intensive Care Units from an Asian Perspective: An Integrative Review of the Research Literature/td>	2019 Exclude	
62548594	Exclude Kim (2020)	Addressing the Needs of Mothers with Infants in the Neonatal Intensive Care Unit: A Qualitative Secondary Analysis/td>	2020 Exclude	
62548604	Exclude Kynoe (2020)	When a common language is missing: Nurse-mother communication in the NICU. A qualitative study./td>	2020 Exclude	

62548559	Loewy (2015) Exclude	NICU music therapy: Song of kin as critical lullaby in research and practice/td>	2015 Exclude	
62548619	Mann (2016) Include ✓	Design, Implementation, and Early Outcome Indicators of a New Family-Integrated Neonatal Unit./td>	2016 Include	✓
62548615	Marfurt-Russenberger (2016) Exclude	The Experiences of Professionals Regarding Involvement of Parents in Neonatal Pain Management./td>	2016 Exclude	
62548646	Mnisi (2019) Exclude	Postnatal depressive features in mothers of neonates admitted to a neonatal unit at Steve Biko Academic Hospital: The role of sociodemographic and psychosocial factors./td>	2019 Exclude	
62548653	Mukunya (2018) Include ✓	"We shall count it as a part of kyogero": acceptability and considerations for scale up of single dose chlorhexidine for umbilical cord care in Central Uganda./td>	2018 Include	✓
62612581	Ngozi (2020) Include ✓	Child Handling Cultural Practices for Neuromotor Development in Infants in a Cohort of African Population: A Prospective Analytical Study/td>	2020 Include	✓
62815570	Nitin et al (2013) Include ✓	Infant Rearing Practices in South India: A Longitudinal Study/td>	2013 Include	✓
62548655	NOVOROĐENAČKA RAZVOJNA... (SIPE) Exclude	NOVOROĐENAČKA RAZVOJNA NJEGA I NADZOR./td>	Exclude	
62548608	Peng (2012) Include ✓	Cultural practices and end-of-life decision making in the neonatal intensive care unit in Taiwan./td>	2012 Include	✓
62548658	Perceptions and Practices... (Sarapat) ✓ Include	Perceptions and Practices of Parents in Caring for their Hospitalized Preterm Infants./td>	Include	✓
62548600	Rosenthal (2013) Exclude	A meta-ethnography and theory of parental ethical decision making in the neonatal intensive care unit./td>	2013 Exclude	
62548631	Sables-Baus (2012) Exclude	An exemplar for evidence-based nursing practice using the Magnet(®) model as the framework for change: oral feeding practice in the neonatal intensive care unit./td>	2012 Exclude	
62548663	Salam (2015) Exclude	Effect of emollient therapy on clinical outcomes in preterm neonates in Pakistan: a randomised controlled trial./td>	2015 Exclude	
62548597	Setiawan (2019) Exclude	Understanding the Effects of Neonatal Early Discharge on Parents: A Literature Review./td>	2019 Exclude	
62548566	Shorey (2016) Exclude	Skin-to-skin contact by fathers and the impact on infant and paternal outcomes: an integrative review/td>	2016 Exclude	
62548602	Skene (2019) Exclude	Developing family-centred care in a neonatal intensive care unit: An action research study./td>	2019 Exclude	
62548609	Thorley (2014) Include ✓	Milk siblingship, religious and secular: History, applications, and implications for practice./td>	2014 Include	✓
62548613	Turner (2014) Exclude	The neonatal nurses' view of their role in emotional support of parents and its complexities./td>	2014 Exclude	
62548593	Umberger (2018) Exclude	Enhancing NICU parent engagement and empowerment/td>	2018 Exclude	
62548670	Upadhyay (2012) Include ✓	Role of Cultural Beliefs in Influencing Selected Newborn Care Practices in Rural Haryana./td>	2012 Include	✓

62548561	Van McCrary (2014) Exclude	A delicate subject: The impact of cultural factors on neonatal and perinatal decision making/td>	2014 Exclude	
62548616	Wiebe (2011) Include ✓	Parent perspectives from a neonatal intensive care unit: a missing piece of the culturally congruent care puzzle./td>	2011 Include ✓	
62548552	Xu (2018) Exclude	Childbirth and Early Newborn Care practices in 4 provinces in China: A comparison with WHO recommendations/td>	2018 Exclude	
62548544	Yue (2020) Exclude	Barriers and facilitators of kangaroo mother care adoption in five Chinese hospitals: A qualitative study/td>	2020 Exclude	
62548562	Zamanzadeh (2013) Exclude	Mothers' experiences of infants discharge in Iranian NICU culture: A qualitative study/td>	2013 Exclude	

Blue text : Studies screened by Madimetja Nyaloko
Orange text : Studies screen by Welma Lubbe
✓ : Studies included for Critical Appraisal
